# The outcome of treatment for anorexia nervosa inpatients who required urgent hospitalization

**DOI:** 10.1186/1751-0759-8-20

**Published:** 2014-09-03

**Authors:** Keisuke Kawai, Sakino Yamashita, Gen Komaki, Miki Shimizu, Megumi Nakashima, Samami Etou, Shu Takakura, Masato Takii, Chiharu Kubo, Nobuyuki Sudo

**Affiliations:** 1 Department of Psychosomatic Medicine, Graduate School of Medical Science, Kyushu University, 3-1-1 Higashi-ku, Fukuoka 812-8582, Japan; 2 School of Health Sciences Fukuoka, International University of Health and Welfare, Fukuoka, Japan; 3 Kyushu University, 3-1-1 Higashi-ku, Fukuoka 812-8582, Japan

**Keywords:** Anorexia nervosa, Urgent hospitalization, Outcome, BMI, MMPI, EDI

## Abstract

**Background:**

This study was done to determine which psychosocial factors are related to the urgent hospitalization of anorexia nervosa patients (AN) due to extremely poor physical condition and to evaluate their outcome after inpatient treatment.

**Methods:**

133 hospitalized AN patients were classified into an urgent hospitalization (n = 24) or a planned hospitalization (n = 109) group. Multiple regression analysis was done of clinical features, body mass index (BMI), psychological tests [The Minnesota Multiphasic Personality Inventory (MMPI), alexithymia, relationship with parents, and the Eating Disorder Inventory (EDI)]. The effectiveness of treatment was prospectively determined two years after discharge by the Global Clinical Score (GCS). The hospitalized weight gain and the frequency of outpatient visits were evaluated.

**Results:**

Of the factors assessed, only BMI at admission was related to the necessity of urgent hospitalization (β = − 1.063, P = 0.00). The urgent group had significantly more weight loss after discharge and poorer social adaptation on the GCS, even when the patient had a sufficient increase in body weight during inpatient treatment and an equivalent number of outpatient consultations.

**Conclusion:**

None of the parameters of the psychosocial tests studied were significantly different between the groups. The outcome of the urgent group was poor. Two years after discharge they had difficulty maintaining weight and continued to have poor social adaptation.

## Background

Anorexia nervosa (AN) is a disease that results in extreme weight loss due to an intense fear of gaining weight, without any relation to organic disease. Most of the pathophysiological complications related to AN are reversible with improved nutritional status; however, some of the physical consequences can be life-threatening [1-4]. Many practice guidelines have been promulgated that indicate factors useful for assessing the need for hospitalization on the day of consultation, such as body weight, temperature, blood pressure, and electrolyte imbalance [5,6].

We recently reported that patients who required urgent hospitalization because of serious physical complications had a significantly more rapid decrease of Body mass index (BMI) as the they got closer to the time of admission than did AN patients for whom admission was planned [7] and that there is no considerable difference in social factors between the groups [7].

However, research is inconclusive as to which psychological factors are related to the necessity for the urgent hospitalization of anorexia nervosa (AN) patients because of serious physical complications or as to their outcome after inpatient treatment.

According to the Ministry of Health, Labor, and Welfare of Japan, about half of all patients are transferred to a specialist by a family doctor (psychosomatic doctor or psychiatrist) and the other half directly visit a specialist. No day care or specialized outpatient system has been established for Japanese eating disorder patients.

With the above in mind, we hypothesized the following: 1. That the psychopathology of AN patients in severe physical condition would be more serious than that of other AN patients, even if their background is equivalent. For example, the drive for thinness is strong, denial or avoidance from their situation is strong, or the relationship with their parents is poor. 2. That the outcome after inpatient treatment would be poor. To confirm these hypotheses, we compared the differences in the psychosocial background, the time course of inpatient treatment, and the outcome of treatment of patients who required urgent hospitalization and AN patients with planned admission.

## Methods

### Participants

The study group was composed of 133 consecutive new AN inpatients at the Department of Psychosomatic Medicine of Kyushu University Hospital between 2002 and 2009. A diagnosis of AN was based on the eating disorders section of the Structured Clinical Interview for DSM -IV Axis I Disorders [8]. Our institution mainly treats patients 13 years or older.

### Clinical characteristics

BMI (kg/m^2^) was calculated as the ratio of body weight (kg) to height (m) squared. The inpatient BMI was measured before breakfast after urination with the patients in similar clothes, usually light pajamas. Serum BUN/Cr was assessed the day after hospitalization as an index of dehydration. All measurements were performed by an experienced technician.

To investigate the clinical factors related to the necessity for urgent hospitalization, we classified the patients into two groups by if their hospitalization was urgent because of extremely poor physical condition or planned.

#### Urgent group

Patients hospitalized because of disturbance of consciousness and/or difficulty walking on the day of consultation. These symptoms are indicators of the need for immediate hospitalization [1,5,6]. All causes of difficulty walking, including muscle weakness, heart failure, and infection, were included (11 cases admitted directly to Kyushu University Hospital, 8 cases transferred from other hospitals soon after urgent hospitalization, and 5 cases with a history of urgent hospitalization referred from other hospitals and treated as outpatients).

#### Planned group (control)

Patients hospitalized for AN other than those who required urgent hospitalization.

The study investigators were blinded to the grouping of the patients (urgent vs. planned group). All measurements were performed by experienced investigators who have ten years or more clinical experience. A study investigator interviewed each of the participants to determine illness duration and medical history (Table 1).

**Table 1:** Clinical characteristics of anorexia nervosa patients who required urgent admission and a planned admission control group

	**Urgent group (n = 24)**	**Planned group (n = 109)**	**p**
Age (years)	28.3 ± 12.41	23.5 ± 8.77	0.104^a^
Sex (male/female)	0/24	7/102	0.24^b^
Duration (years)	8.45 ± 8.75	5.73 ± 6.61	0.165^a^
History of admission(+/−)	11/13	36/73	0.225^b^
Subtype (AN-R/AN-BP)	13/11	53/56	0.395^b^
BMI at admission (kg/m^2^)	11.11 ± 1.48	13.91 ± 1.92*	0.00^a^

Values are expressed as mean ± SD or number of patients: Comparisons among groups were made using the Unpaired student’s t test (a) and Chi-square test (b).

AN-R: Anorexia nervosa Restricting type, AB-BP: Anorexia nervosa Binge-Purging type, BMI: Body Mass Index. *P < 0.05.

### Socio-demographic and psychological measures at admission

Differences in the socio-demographic variables (Table 2) and the psychological features of the groups were examined. In this study, we added two socio-demographic factors, living alone and history of bullying or abuse, to the items of our previous study [7]. The following psychological tests were given at the time of admission (within one month of hospitalization).

**Table 2:** Socio-demographic variables of AN patients who required urgent admission and a planned admission control group

	**Urgent group (n = 24)**	**Planned group (n = 109)**	**p**
Married or with partner	9(37.5%)	15 (13.8%)	0.021*
Living alone	2(8.3%)	22(20.2%)	0.417
Divorced parents	3(1.5%)	9(8.2%)	0.371
History of bullying or abuse	8 (33.3%)	27(24.8%)	0.267
Education level			
High school, vocational school	16(66.7%)	76(69.7%)	0.472
College or more	2(8.3%)	16(14.7%)	0.327
Employed	0(0%)	16(14.7%)	0.033*
A history of regular employment	7(29.1%)	21(19.3%)	0.208
A history of part-time work	8(33.3%)	44(40.4%)	0.345
Lifestyle -related factors			
Smoking	4(16.6%)	24(22.0%)	0.345
Social drinking or more	4(16.6%)	36(33.0%)	0.385

Values are expressed as number of patients (%). Comparisons among groups were made using the chi-square test. *P < 0.05.

The MMPI (The Minnesota Multiphasic Personality Inventory) was used to evaluate the personality and general psychopathology of the patients [9]. Three validity and 10 clinical scales were scored.

The TAS − 20 (Toronto Alexithymia scale 20) is a 20-item instrument that is one of the most commonly used measures of alexithymia [10,11]. Alexithymia refers to people who have difficulty identifying and describing emotions, tend to minimize emotional experience, and focus their attention externally. The TAS-20 consists of three subscales: Difficulty Describing Feelings (F1), Difficulty Identifying Feelings (F2), and Externally-Oriented Thinking (F3).

The PBI (Parental Bonding Instrument ) is a self-report measure of the child-rearing attitude of parents as seen by their children. The PBI consists of four subscales: Mother care (M-CA), Mother overprotection (M-OP), Father care (F-CA), and Father overprotection (F-OP) [12].

The EDI (Eating disorder inventory) is a diagnostic tool designed for use in a clinical setting to assess the presence of an eating disorder [13]. It is comprised of 64 questions divided into eight subscales (Drive for thinness (DT), Interoceptive awareness (IA), Bulimia (B), Body dissatisfaction (BD), Ineffectiveness (I), Maturity fears (MF), Perfectionism (P), and Interpersonal distrust (ID)) (Table 2) [13].

Multiple regression analysis of age, sex, duration of AN, BMI, BUN/Cr, social circumstances, and psychological factors was done to determine their influence on the necessity of urgent hospitalization.

Outcome of inpatient treatment (Assessment of AN severity before hospitalization and two years after discharge): Our AN patients are treated with an inpatient therapy we call the “cognitive behavioral approach with behavioral limitation” [14]. This therapy follows the guidelines of the Japanese treatment manual and is based on operant behavioral therapy and cognitive behavioral therapy (CBT). The length of hospitalization, BMI at discharge, speed of hospitalized weight gain, and the frequency of nasal feeding were compared. After discharge, regular visits were required. Weight and height were measured in our outpatient clinic with the patient wearing light dress with no jacket. Neither the time of meals nor the presence or absence of meals on the day of measurement were evaluated. However, confirmation was done to insure that the patients did not hide weights in their clothes pockets. The frequency of outpatient visits was compared for two years after discharge. The criteria for readmission depended on an agreement with the individual patient, but basically were weight loss of 3-4 kg or the reappearance of difficulty in daily life.

The outcome of the urgent and planned groups was based on comparison of data collected before and during hospitalization and two years after discharge. Assessment of AN severity was based on the Global Clinical Score (GCS) [15]. GCS measures the following on a 0–3 scale: Body weight, eating habits (selective restriction in the variety of the diet), vomiting, over eating, laxative abuse, social adjustment, and educational and/or vocational adjustment. Menstrual status is scored 0–2. Total scores of 0 to 3 were defined as “excellent”, 4 to 7 “much improved”, 8 to 11 “symptomatic”, and above 11 “poor”. In addition, the subscales of GCS were evaluated to determine factors possibly associated with poor outcome. Patients were excluded who had missing data on the GCS, discontinued outpatient treatment, or dropped out of their treatment program at another hospital and for whom the required data was unavailable.

All participants and/or their parents provided written informed consent at the time of admission. The study was approved by the Kyushu University Research and Ethics Committee.

### Data analysis

All analyses were performed using SPSS for Windows ver.14.0 J. Results are presented as means ± standard deviation (SD). Comparisons among groups were made using the unpaired t-test and Wilcoxon signed-rank test. Categorical data were analyzed using Pearson’s chi-square test. In the analysis of MMPI scores, univariate methods would tend to over-represent the number of between group differences because the scales within the MMPI are generally significantly correlated with one-another. In this study, we therefore chose multivariate analysis to determine between group differences in the MMPI clinical scales, content scales, and critical items. Multivariate analysis of variance (MANOVA) was done to determine possible between group differences in mean scale data. The criterion for statistical significance was p < 0.05. When there was a significant statistical difference between the groups, the data were further analyzed using residual analysis (multivariate linear regression analysis).

## Results

### Clinical characteristics

The urgent group had a significantly lower BMI than the planned group at first visit (p = 0.00 Table 1). The average age was older and the duration of illness of the urgent group was longer than those of the planned group, but neither reached significance. Comparison of the urgent and planned groups found no difference in sex, history of admission, or type of AN. The BUN/Cr of the urgent group was significant higher than that of the planned group (36.7 ± 18.3, n = 24 , vs, 21.51 ± 9.08 n = 109 P = 0.00).

### Socio-demographic variables

None of the main social background factors; living alone, divorce of the parents, history of suffering bullying or abuse, educational level, employment history, smoking, or alcohol consumption; were different between the groups. The percentage who were married was high (P = 0.021 Table 1) in the urgent group. The urgent group had a significantly lower rate of employment before admission (P = 0.033 Table 2).

### Psychological variables

**Figure 1:**
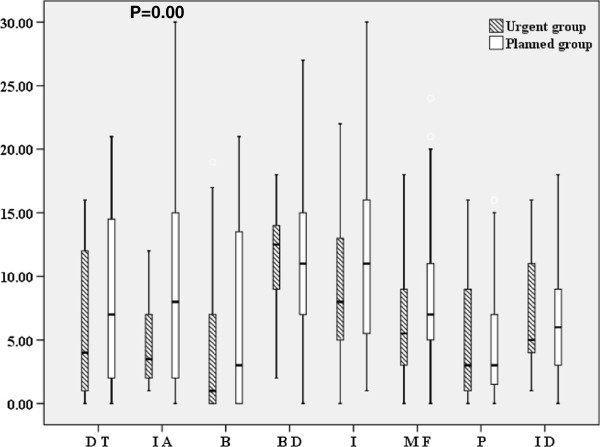
**EDI scores of the urgent and planned admission groups at admission.** *P = 0.00 (urgent vs. planned group: IA) Boxes encompass those patients whose EDI were within the second and third quartiles of the respective group; the vertical line within a box represents the median EDI for the respective group. Urgent group: Anorexia nervosa requiring urgent admission due to disturbance of consciousness or difficulty walking (n = 18); Planned group: Anorexia nervosa, excepting those who required urgent hospitalization (a planned admission control group: n = 99). EDI; Eating disorder inventory, DT: Drive for thinness, IA: Interoceptive awareness, B:Bulimia, BD: Body dissatisfaction, I:Ineffectiveness, MF: Maturity fears, P:Perfectionism, ID: Interpersonal distrust.

MMPI was taken by112 of the 133 patients. Fifteen patients with a scale score of higher than 55 points were excluded because the validity of the test was suspect. No significant between group differences were found for any of the items of the MMPI by MANOVA (Wilks’ lambda = 0.665,F (31,53) = 0.862, P = 0.666). TAS was taken by102 of the 133 patients. No significant differences between the urgent and planed groups were found for F1, F2, F3, or total score (17.82 ± 6.52 vs. 19.72 ± 7.13, 17.94 ± 4.47 vs. 17.24 ± 4.04, 19.76 ± 4.42 vs. 19.42 ± 4.08, 55.23 ± 10.79 vs. 56.64 ± 10.02). PBI was taken by 110 of the 133 patients. No significant between group differences were found in M-CA, M-OP, F-CA, or F-OP (24.60 ± 8.23 vs. 25.94 ± 8.68, 12.56 ± 9.68 vs. 13.05 ± 8.41, 23.0 ± 3.64 vs. 23.3 ± 10.57, 9.55 ± 6.02 vs. 11.20 ± 8.06). EDI (Figure 1) was taken by 110 of the 133 patients. No significant between group differences were found in Drive for thinness, Bulimia, Body dissatisfaction, Ineffectiveness, Maturity fears, Perfectionism, or Interpersonal distrust (6.06 ± 5.79, vs. 8.19 ± 6.89, 4.17 ± 5.98 vs. 6.74 ± 7.39, 11.1 ± 4.66 vs. 11.29 ± 5.56, 9.39 ± 5.97 vs. 11.73 ± 7.47, 7.22 ± 5.16 vs. 8.13 ± 4.90, 4.89 ± 4.72 vs. 4.59 ± 4.34, 7.50 ± 4.76 vs. 6.38 ± 3.92). The interoceptive awareness of the urgent group was lower than that of the planned group (4.67 ± 3.61 vs. 9.33 ± 8.12, P = 0.00). The total score of the urgent group was lower, without significance, than that of the planned group (55.72 ± 21.06 vs. 66.44 ± 34.79: P = 0.086). Standardized correction of the MMPI test was done by MANOVA. Other psychological factors were compared with two tests. Standardized correction was insured by setting the P value at 0.00.

Multiple regression analysis was done of factors possibly related to the necessity of urgent hospitalization. Factors that demonstrated a P value of 0.1 or less, such as BMI, BUN/Cr, age, marital status, employment status, and interoceptive awareness were input for analysis by the forced elimination method. Only BMI at admission was negatively correlated with urgent hospitalization (β = − 1.0630, P = 0.00).

### Outcome of inpatient treatment

The length of hospitalization of the urgent group (193.46 ± 86.8 days) was significantly longer than that of the planned group (140.80 ± 96.34, P = 0.015). The BMI at discharge of the urgent group (15.79 ± 2.22 kg/m^2^) was not different than that of the planned group (16.49 ± 2.05, P = 0.13). For the comparison of the increase of BMI during the treatment of patients hospitalized two weeks or more, the planned group consisted of 104 patients and the urgent group 24. During hospitalization, the speed of BMI increase per week of the urgent hospitalization group (0.195 ± 0.115 kg/m^2^/week) was significantly faster than that of the planned group (0.098 ± 0.159, P = 0.005).

The frequency of nasal feeding of the urgent group was significantly higher than that of the planned group (18/24 vs. 55/106, P = 0.04). The frequency of outpatient visits was not significantly different (urgent group 24.5 ± 11.16 n = 18 vs. planned group 20.65 ± 10.4 n = 71, P = 0.458).

**Figure 2:**
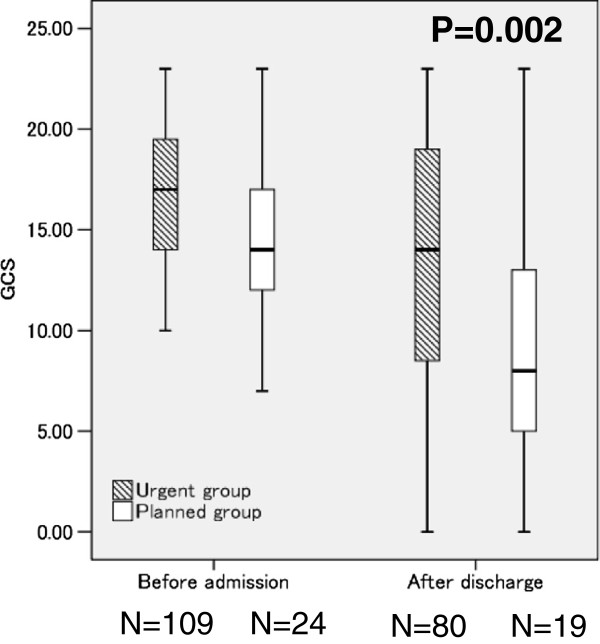
**The outcome of inpatient treatment: Comparison of parameters before admission and two years after discharge.** *P = 0.002 (urgent vs. planned group: two years after discharge) Boxes encompass those patients whose GCS were within the second and third quartiles of the respective group; the vertical line within a box represents the median GCS for the respective group. Urgent group: Anorexia nervosa requiring urgent admission due to disturbance of consciousness or difficulty walking (n = 19); Planned group: Anorexia nervosa, excepting those who required urgent hospitalization (n = 80). GCS: Global Clinical Score.

In the GCS given before admission, no overall difference in the groups was found (15.62 ± 3.3 vs. 14.75 ± 3.5, P = 0.255: Figure 2). However, body weight, menstrual status, and educational and/or vocational adjustment of the urgent group were significantly poorer than was found for the planned group (Table 3).

**Table 3:** GCS subscales for anorexia nervosa patients who required urgent admission and a planned admission control group

	**Urgent group**	**Planned group**	**p**
**Before admission**	(n = 24)	(n = 109)	
Body weight	3 ± 0	2.87 ± 0.41	0.01*
Eating habits	5.33 ± 2.82	5.62 ± 2.66	.65
Menstrual state	2.0 ± 0	1.83 ± 0.48	0.01*
Social adjustment:	2.58 ± 0.88	2.40 ± 0.85	0.369
Educational and/or vocational adjustment	2.70 ± 0.81	2.02 ± 1.29	0.01*
**After discharge**	(n = 19)	(n = 81)	
Body weight	2.11 ± 1.15	1.43 ± 1.11	0.03*
Eating habits	5.42 ± 4.56	3.73 ± 3.04	0.138
Menstrual state	1.21 ± 0.97	0.86 ± 0.90	0.169
Social adjustment	2.42 ± 0.90	1.74 ± 1.07	0.08*
Educational and/or vocational adjustment	2.26 ± 1.24	1.15 ± 1.28	0.002*

Values are expressed as mean ± SD Comparisons among groups were made using the Wilcoxon signed-rank test. *P < 0.05.

The data of 81 of the 109 patients in the planned group and 19 of the 24 in the urgent group were available for comparison two years after discharge (Figure 2). The urgent group had a significantly poorer outcome than the planned group (13.42 ± 7.39 ‘poor’ vs. 8.91 ± 5.17, ‘symptomatic’ , P = 0.002). In the comparison of the subscales of the GCS, the body weight, social adjustment, and educational and/or vocational adjustment of the urgent group were significantly poorer than was found for the planned group (Table 3). In the urgent group, 3 (15.7%) participants were categorized as excellent, 2 (9.5%) much improved, 1 (5.3%) symptomatic, and 13 (68.4%) poor two years after discharge. None of the patients in the urgent group required re-hospitalized because of disturbance of consciousness or difficulty in walking during the two years after discharge. In the planned group, 12 (14.8%) participants were categorized as excellent, 23 (23.4%) much improved, 22 (27.2%) symptomatic, and 24 (29.6%) poor. The BMI of the urgent group two years after the discharge was significantly lower than that of the planned group (15.44 ± 3.65 vs. 17.82 ± 3.65, respectively, P = 0.001).

## Discussion

Multiple regression analysis of the clinical features and psychosocial aspects of AN patients extracted only BMI at admission as related to the necessity of urgent hospitalization because of extreme physical condition. Contrary to our expectations, the compatibility of all of the psychological items of MMPI, TAS-20, and PBI between the two groups was surprising. The drive for thinness and denial or avoidance of their situation was the same for both groups. Furthermore, the relationship with their parents, who are important in the decision for hospitalization, was the same in both groups. Even though the patients had a sufficient increase in body weight during inpatient treatment, the outcome of the urgent group was poor. The patients were asked to fill in their questionnaires within one month of hospitalization and after stabilization, especially for the urgent group. Some of the initial differences between the groups may have been affected by bias related to the longer period of time before filling in the questionnaires of the urgent group.

We highlight possible mechanisms related to the necessity of urgent hospitalization and the poor outcome of the urgent group. First, new findings of this paper are that the outcome of the urgent group is worse than that of the planned group and that the urgent group continued to have a significantly low level of body weight and poor social and educational and/or vocational adjustment two years after inpatient treatment. In the introduction, we hypothesized that the psychological background of the two groups would be different. None of the common psychosocial factors studied, including psychological personality and family support, were related to the urgent hospitalization of these AN patients. Nevertheless, the patients in the urgent group had difficulty retaining the body weight gained during hospitalization and in adapting to daily life. The common psychological tests may lack sensitivity for detecting these characteristics. However, the results suggest that difficulty in social adaptation is an important factor in inducing secondary low weight as an avoidance behavior. It will be necessary to develop new assessment tools that can more accurately evaluate the desire for thinness as it relates to denial and difficulty with social adaptation.

The second important finding was that a low BMI was an important factor in the necessity of urgent hospitalization. We previously reported that the speed of BMI reduction increases when BMI becomes low (<13-14 kg/m^2^) [16]. When BMI is lower than 13-14 m/kg^2^ the supply of energy changes from fat mass to protein [7,16]. In fact, one gram of fat mass (9 kcal/g) has about twice the calories of protein (kcal/g). Low BMI itself seems to be related to a further reduction. Hyperactivity is an important core psychopathological trait of anorexia nervosa patients [17]. Compared to healthy subjects, patients and athletes rated exercise stimuli as more pleasant [17]. Recently, a significant subgroup of high-level AN-exercisers (66%) with increased energy requirements was identified [18]. The rate by subtype of AN did not differ in this study but there may be a difference in the degree of activity of the urgent and planned groups. For AN in general, early age at onset of illness, young age, and short duration of illness have been associated with a good outcome, and low body weight, vomiting, bulimia, purgative abuse, and psychiatric comorbidities with a poor outcome [1-3]. It was reported that the prognosis of the AN group with BMI less than 13 kg/m^2^ at referral was poor [7,19]. Low BMI would seem to be a strong contributor to the poor outcome of patients who require urgent hospitalization.

Finally, we previously reported that BMI was inversely correlated with maturity fears in a multivariable analysis (β = −0.375, P = 0.046) of EDI [20]. The current study did not find maturity fears to be a significant factor. The psychological mechanism (s) that led to the necessity of urgent hospitalization cannot be explained simply by the association between low BMI and the EDI score. In addition, our review of previous research on whether or not the severity of the physical condition of AN patients is related to genetic and/or biological factors identified studies of eating, weight, and energy consumption-related genetic polymorphism [21-23]. However, the relation between the genetic polymorphisms of energy consumption peptides and the appetite control of AN patients is still inconclusive [21-23]. Genetic factors related to an excessive decrease of weight may become apparent in the future.

The GCS status of the urgent group remained “poor” two years after discharge. However, it is important that patients who require urgent hospitalization have long-term survival after treatment, without the need for urgent re-hospitalization. The general condition of our patients was so severe that their mean BMI was in the lowest category used in previous follow-up studies [14]. Our treatment was successful as the first step in a regimen that led to a good course for the patients of the urgent group. From a psychosocial aspect, AN patients in extremely poor physical condition are similar to other AN patients. It has been proposed that various psychotherapies and nutrition therapies for maintaining weight are required in the treatment of AN [1,4,5]. Patients who require urgent hospitalization need special programs for weight recovery and strong social support in addition to the usual treatment protocols.

The patients in the urgent group may have lacked motivation to accept the treatment regimen because their hospitalization was not of their own volition. However, the urgent group was significantly faster than the planned group in the average speed of increase in body weight. Certainly, the degree of the dehydration of the urgent group was significantly greater than that of the planned group. Also, the frequency of nasal feeding of the urgent group was significantly higher that of the planned group. These points may be related to the difference in weight gain. Although these points deserve consideration, the urgent group seemed to take their inpatient treatment seriously. The frequency of outpatient visits did not significantly differ between the groups. Future study will be required to clarify the role of motivation in the success of treatment.

For adult AN outpatients, there are promising findings for focal psychodynamic psychotherapy as well as cognitive behavior psychotherapy [24]. However, a clear limitation for use in an outpatient approach is that the international guidelines for weight refer to a weight greater than 15.0 kg/m^2^. Japanese in general tend to be lighter than Europeans or Americans. It has been recommended by the Ministry of Health, Labor, and Welfare of Japan that doctors use lower than 55% of the national standard weight (BMI 12.1 kg/ m^2^) as an index for the urgent hospitalization of AN patients., Taking the above into account, some of the urgent group patients may have difficulty in their psychological therapy after a discharge because of their low weight, which might affect the prognosis of the urgent group.

### Limitations

This research was done in only one institution. Study including more institutions will be required in the future. Another limitation is that the follow-up was only two years, which might be too short to determine the prognosis. Also, the mean waiting time before admission for inpatient care was not calculated. No significant differences in GCS were found between the urgent and planned groups before admission. GCS has weaknesses in its scoring that makes it more difficult to distinguish relevant factors in terms of outcome than other measures such as the Morgan Russell score or the eating disorder examination (EDE) which use expert interviews to give more valid outcome scores and that might be useful for this type of investigation [25,26]. It is possible that episodes of malnutrition cause damage and changes in brain function.

It will be necessary to do further study to assess the role of brain function on cognitive impairment as it relates to the prolonged malnutrition and/or extremely poor physical condition of AN patients.

## Conclusions

None of the parameters of the characteristic psychosocial tests studied were related to the AN of patients who required urgent hospitalization due to extremely poor physical condition. Such patients were shown to have difficulty in the recovery of body weight and in improving social adjustment. The outcome of AN patients who required urgent hospitalization was poor.

## Competing interests

The authors declare that they have no competing interests.

## Authors’ contributions

KK conceived this study and performed the study design and the statistical analysis. SY, KG, and ST participated in the study design and helped draft the manuscript. SM, SE, MN, and MT assisted with the study. CK and NT contributed to revision of the manuscript. All authors read and approved the final manuscript.
